# Multiple instance learning for differentiating neoplastic and non-neoplastic brain lesions using MR spectroscopy

**DOI:** 10.1016/j.ynirp.2026.100418

**Published:** 2026-10-17

**Authors:** Iskra Gacheva, Diyuan Lu, Gerhard Kurz, Jochen Triesch, Elke Hattingen, Nenad Polomac

**Affiliations:** a Institute for Neuroradiology, University Hospital Frankfurt, Frankfurt am Main, 60528, Germany; b Frankfurt Institute for Advanced Studies (FIAS), Frankfurt am Main, 60438, Germany; c Independent Researcher, Germany

**Keywords:** Multiple instance learning, MR spectroscopy, Brain tumors, Neuroradiology

## Abstract

**Objective:**

To evaluate whether magnetic resonance spectroscopy (MRS) combined with multiple instance learning (MIL) can differentiate neoplastic from non-neoplastic brain lesions by analyzing full spectral patterns rather than isolated metabolite peak ratios. We hypothesized that treating the full spectrum as a metabolic fingerprint, captured through deep learning, would enable more robust tissue classification than conventional single-peak analysis.

**Methods:**

We retrospectively analyzed MRS data from 422 patients (7442 spectra total): 6598 spectra for training and 844 spectra in the independent internal hold-out test set. Classification was formulated as a MIL problem to address patient-level weak supervision and variable bag sizes. A deep neural network was trained with data augmentation and evaluated with two permutation-invariant aggregation strategies: min–max–mean pooling and attention-based pooling. Diagnostic performance was benchmarked against experienced neuroradiologists.

**Results:**

The best-performing MIL configuration, combining a convolutional neural network (CNN) with 3Pool aggregation and data augmentation (CNN-3Pool + DA), achieved an AUC of 0.82 with a sensitivity of 0.76 and specificity of 0.80 on the independent hold-out test set. In the MRS-only reader comparison, the participating neuroradiologists showed an average sensitivity of 0.54 and specificity of 0.88. Attention-based MIL additionally enabled visualization of the relative contribution of individual spectra, highlighting clinically established spectral features, including lipid and choline peaks, as well as the Glx region (2.2–2.4 ppm).

**Conclusions:**

Formulating MRS-based brain tumor classification as a MIL problem enables robust learning from heterogeneous, weakly labeled clinical data. The attention mechanism provides interpretable insights into spectral decision-making and highlights candidate biomarkers for future validation. This framework offers a scalable and interpretable approach to clinical MRS analysis.

## Highlights

• Multiple instance learning for patient-level MRS classification.
• Aggregates voxel spectra into a single diagnostic decision.
• Attention highlights clinically relevant spectral regions.
• Robust tumor detection on independent validation data.
• Complements neuroradiologists with higher sensitivity.

## Graphical abstract

**Figure:**
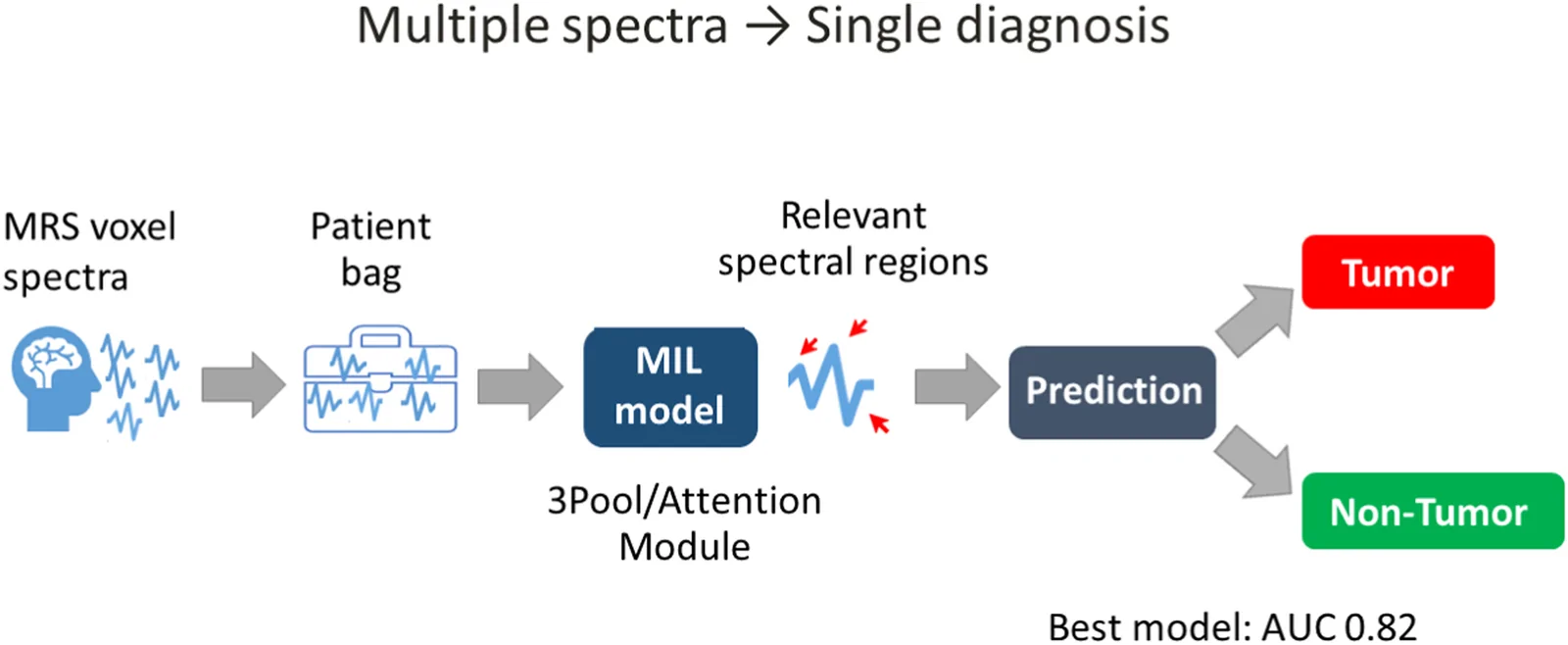

## 1 Introduction

Brain tumors are among the most serious neurological diseases, and early, accurate diagnosis is critical for treatment planning and outcome (Louis et al., 2007; Weller et al., 2017). Conventional magnetic resonance imaging (MRI) is the standard for detection and follow-up, but it often cannot reliably distinguish gliomas from non-neoplastic lesions or treatment-related changes, as these entities may present with similar imaging features (Villanueva-Meyer et al., 2017). Differential diagnoses that can mimic gliomas include inflammatory lesions, demyelinating diseases, vascular lesions, developmental abnormalities such as focal cortical dysplasia, and, more rarely, enlarged Virchow–Robin spaces (Modha et al., 2000; Kwee and Kwee, 2007; Raab et al., 2008). These conditions may closely resemble neoplasms on MRI, complicating interpretation in everyday clinical practice (see Fig. 1).

**Fig. 1:**
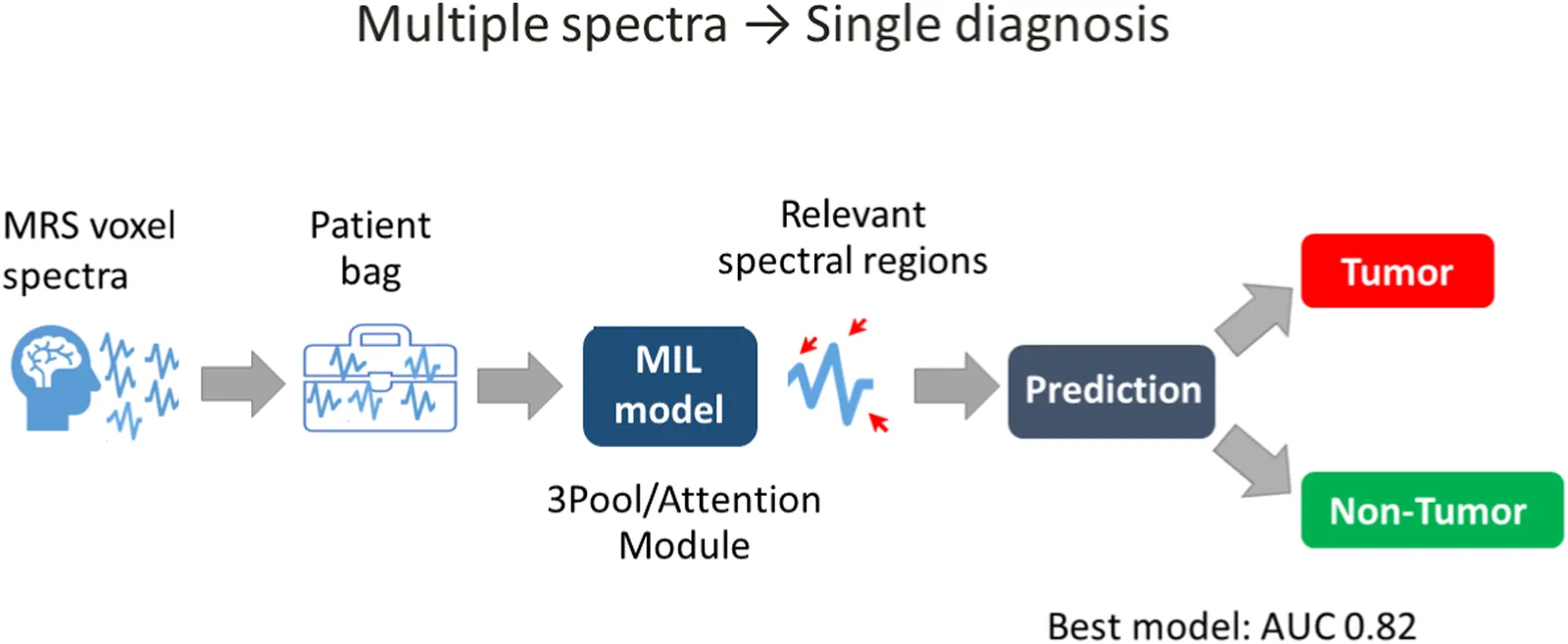
Workflow of the multiple-instance learning (MIL) approach. For each patient, multiple MRS spectra were grouped into a bag. A deep neural network extracted spectral features, which were aggregated either by **3Pool** (min–max–mean) or by an **attention** module to obtain a permutation-invariant patient representation, followed by a softmax classifier (tumor vs. non-tumor). Illustration adapted from Lu et al. (Lu et al., 2021a).

MRS provides metabolic information complementary to structural imaging (Raab et al., 2008; Ditter and Hattingen, 2017). Conventionally, neuroradiologists either assess individual metabolite concentrations (e.g., choline, N-acetylaspartate (NAA), lactate) (Setzer et al., 2007) or interpret the entire spectrum as a pattern, which better reflects clinical practice but is complex, subjective, and difficult to reproduce (Hattingen et al., 2009; Law et al., 2002). Consequently, diagnostic accuracy remains limited, especially in the presence of artifacts, variable tumor localization, or examiner-dependent variability (Posti et al., 2015).

Machine learning (ML) has the potential to overcome these limitations by automatically extracting relevant features and identifying spectral patterns beyond single metabolite peaks (Erickson et al., 2017; Choy et al., 2018). However, clinical MRS data present specific challenges: labels are only available at the patient level rather than the voxel level, annotations are often inconsistent, and the number of spectra per patient is highly imbalanced (Lu et al., 2021a). Multiple instance learning (MIL) is particularly well suited to addressing these issues, as it allows training on heterogeneous, weakly labeled data (Dietterich et al., 1997; Carbonneau et al., 2018).

MIL is a weakly supervised learning framework in which labels are assigned to groups of instances (“bags”) rather than to each individual instance (Dietterich et al., 1997). This approach is particularly useful when reliable instance-level annotations are difficult to obtain. In the present study, spectra from a given patient were therefore grouped into patient-level bags carrying the corresponding diagnostic label. A central requirement when processing such bags is permutation invariance, as the patient-level prediction should not depend on the order of the individual spectra. To achieve this, we investigated two aggregation strategies: a 3Pool module combining minimum, maximum, and average pooling, and an attention-based module assigning different weights to individual instances (Ilse et al., 2018). Compared with fixed pooling, attention-based aggregation can learn the relative contribution of individual instances and thereby provide information on which spectra contribute most strongly to the bag-level representation. In contrast, 3Pool uses predefined minimum, maximum, and mean operations and therefore does not generate instance-specific attention weights. Data augmentation was additionally employed to increase the diversity of the available training data and support model generalization. Although data augmentation can increase training-set diversity and improve generalization, augmentation strategies are domain-dependent and should preserve class-relevant information.

The aim of this study was therefore to evaluate whether an MIL model applied to MRS spectra can improve diagnostic accuracy in distinguishing gliomas from non-neoplastic brain lesions. Our focus was on leveraging spectral patterns rather than isolated metabolites, and on testing robustness under real-world clinical conditions, with comparison against expert neuroradiological assessment.

The present study makes three contributions. First, we conceptualize the full MRS spectrum as a metabolic fingerprint, moving beyond conventional single-peak analysis. Second, we formulate the classification task as a MIL problem to address patient-level weak supervision and variable bag sizes inherent in clinical data. Third, we demonstrate model interpretability through attention-based visualization, identifying spectral regions of diagnostic relevance including previously underutilized metabolite ranges. To our knowledge, this is the first study to apply MIL to clinical MRS data under real-world weak supervision conditions with direct comparison to human expert readers under controlled conditions.

## 2 Materials and methods

### 2.1 Patient cohort

A total of 1371 ^1^H-MRS datasets acquired at our institution between January 2009 and March 2019 were retrospectively evaluated for eligibility. Patients were included if they had a T2-weighted hyperintense brain for which the conventional MRI does not allow a definitive diagnosis to be established. That could not be unequivocally diagnosed based on conventional MRI sequences. Exclusion criteria comprised were as follows: Age below 11 years, metabolic or genetic white matter disorders, cerebral metastases, lymphomas, tumors of non-glial or non-glioneuronal origin with known disease-specific metabolic profiles, and insufficient clinical information to establish a definitive diagnosis. After application of the selection criteria and quality-control procedures, the final study cohort comprised 422 patients (220 female and 202 male; mean age ± standard deviation: 45.5 ± 18.0 years), including 266 patients with brain tumors and 156 with non-neoplastic brain lesions. A total of 7442 spectra were available, comprising 4054 tumor and 3388 non-tumor spectra.

This retrospective study was approved by the Ethics Committee of University Hospital Frankfurt and the Institutional Review Board of the University Cancer Center Frankfurt (UCT) (project number SNO-9-2019; approval date: 16 September 2019). The study included previously acquired ^1^H-MR spectroscopy data obtained during routine clinical evaluation between January 2009 and March 2019. Patient data were provided by the UCT and exported in anonymized form from the institutional PACS archive. The requirement for individual informed consent was waived owing to the retrospective study design and the exclusive use of routinely collected, anonymized clinical data. No patient contact or individual re-identification was involved. Information on ethnicity and socioeconomic status was not available in the retrospective dataset.

### 2.2 Data acquisition

All examinations were performed on a 3T MRI scanner (Skyra, Siemens Healthineers, Erlangen, Germany). The imaging protocol included T2-weighted turbo spin echo imaging in three orthogonal planes for anatomical orientation and two-dimensional proton chemical shift imaging (^1^H-CSI) using a PRESS sequence for metabolite assessment. Spectra were acquired with a voxel size of 13 × 13 × 10 mm^3^, TE = 30 ms, TR = 1500 ms, flip angle = 90°, field of view = 208 × 208 mm, matrix size = 16 × 16, and an acquisition time of 6 min 11 s. Data were stored in DICOM format, anonymized, and subsequently exported for analysis.

### 2.3 Preprocessing and data augmentation

Preprocessing of the spectra included removal of irrelevant information from the DICOM images (letters, baseline, integral values) and uniform scaling of the signal intensities on the y-axis. The x-axis (chemical shift in ppm) was identical across subjects and remained unchanged. Each spectrum was stored as a row vector (1 × 288).

Magnetic resonance spectroscopy data are highly susceptible to various artifacts that can result in corrupted spectra. To ensure that only high-quality data were included in the downstream analysis, we selected two exemplary reference spectra: one from verified tumor tissue and one from normal-appearing white matter. The anatomical and pathological identity of these two tissue regions was independently confirmed using complementary MRI sequences in addition to direct visual inspection of the spectra. To guarantee the robustness of the dataset, a filtering threshold of Pearson's r ≥ 0.5 was implemented. This cutoff was selected because a correlation coefficient of 0.5 represents the transition into a moderate-to-strong effect size, ensuring that the reference spectrum accounts for a minimum of 25% of the shared variance (R^2^ ≥ 0.25) in the retained spectra. Spectra falling below this threshold were excluded to eliminate weak, noisy, or spurious linear relationships lacking predictive relevance.

To improve model robustness, simple data augmentation techniques were applied. These included repetition and resampling of spectra within the labeled patient groups (bags), thereby increasing the number of training examples and supporting better generalization of the machine learning models*.* Specifically, the number of spectra per patient was tripled to balance the dataset and enhance training stability. This approach corresponds to oversampling within patient-level bags to mitigate class imbalance. For a detailed explanation, please refer to our methodological paper (Lu et al., 2021b).

### 2.4 Machine learning framework

In the MIL framework, each patient was represented as a bag containing multiple MRS spectra (instances). Each spectrum within a bag was processed by the same neural network to extract a feature representation. The resulting instance-level features were then combined into a single patient-level representation using a permutation-invariant aggregation module. Two aggregation strategies were evaluated: 3Pool, which combines the minimum, maximum, and mean feature values across the spectra within a bag, and attention-based pooling, which assigns learned weights to individual spectra before aggregation according to the approach proposed by Ilse et al. (2018). The resulting patient-level representation was subsequently passed to a softmax classifier to obtain the probability of a neoplastic versus non-neoplastic lesion. Thus, the model was trained using the diagnosis available at the patient level without requiring separate diagnostic labels for each individual spectrum.

During training, patient-level bags were generated by randomly sampling spectra with replacement from the spectra available for each patient. The number of generated bags per patient was set to three times the number of individual spectra available for that patient. During testing, data augmentation was disabled, and spectra were repeated only when necessary to complete a bag.

We combined these aggregation methods with three types of neural networks: a multilayer perceptron (MLP), a convolutional neural network (CNN) based on Hatami et al. (2018), and a simplified Inception model (Szegedy et al. (2015). These models were simplified compared with typical image-based architectures to account for the lower dimensionality of the MRS data. Further technical details of the ML framework and network architectures have been described previously (Lu et al., 2021a).

All machine-learning experiments were implemented in Python 3 using TensorFlow; preprocessing of spectra was performed in MATLAB (MathWorks, Natick, MA, USA).

### 2.5 Validation strategy

The networks were trained from randomly initialized weights using the Adam optimizer with default parameters (β1 = 0.9, β2 = 0.999), a mini-batch size of 32, and 30 training epochs. The number of spectra per bag (M) was empirically evaluated by varying the bag size from 1 to 51 spectra. Based on these experiments, M = 31 was used as the default configuration.

Model performance was evaluated using a two-step validation strategy. First, five-fold leave-subjects-out cross-validation was performed at the patient level in the development cohort of 383 patients (6598 spectra). Patients were randomly permuted and partitioned into five fixed folds comprising 75, 75, 75, 75, and 83 patients, respectively. The patient and spectrum counts and class distributions of the individual folds are provided in Supplementary Table S2. In each cross-validation iteration, one fold was withheld for testing, while the patients in the remaining four folds were divided at a 4:1 ratio into training and validation sets. All spectra and generated bags belonging to a given patient remained within the same partition, ensuring that no patient contributed data to more than one of the training, validation, or test sets within a given iteration. The validation set was used only to monitor training dynamics, including validation loss and accuracy across epochs. Data augmentation was disabled during testing.

Second, an independent internal hold-out test set comprising 844 spectra from approximately 42 patients was completely withheld from training and validation and was used for comparison with human readers. The test set was divided into eight subsets, each of which was assigned to one of eight neuroradiologists with varying levels of experience in ^1^H-MRS. Readers classified the spectra based solely on the spectral information, without access to structural MRI or clinical information. Model performance was calculated separately on the corresponding subset evaluated by each reader and subsequently averaged across the eight subsets. A fixed probability threshold of 0.5 was used to convert the aggregated patient-level model output into a binary tumor/non-tumor prediction.

Performance metrics included area under the receiver operating characteristic curve (AUC), sensitivity, specificity, accuracy, precision, and F1-score. The 95% confidence interval for the AUC on the independent internal hold-out test set was estimated using nonparametric bootstrap resampling with 2000 iterations and the 2.5th and 97.5th percentiles of the bootstrap distribution.

Model training was performed on a Windows-based workstation equipped with an Intel Core i7-4770 CPU, 16 GB RAM, and an NVIDIA GeForce GTX 1060 GPU with 6 GB memory. Training required less than 3 min for 30 epochs.

## 3 Results

### 3.1 Overall model performance

Among the evaluated MIL configurations, the convolutional neural network combined with 3Pool aggregation and data augmentation (CNN-3Pool + DA) achieved the highest overall performance in five-fold cross-validation (Table 1). On the independent internal hold-out test set of 42 previously unseen patients (844 spectra), this model achieved an AUC of 0.82 (95% CI: 0.78–0.86), correctly identifying 76% of tumor cases (sensitivity) and 80% of non-tumor cases (specificity). Approximately six spectra per patient were sufficient for stable classification, with minimal performance gain from larger sets.

**Table 1:** Performance metrics for different models evaluated on MRS data from 422 patients. Cross-validation results (mean ± SD across five folds) are shown for all machine learning models. Neuroradiologist performance is presented separately for comparison. For the best-performing model (CNN-3Pool + DA), sensitivity and specificity on the independent internal hold-out test set are reported using a fixed probability threshold of 0.5. ^*a*^*Neuroradiologist performance (AUC 0.68, accuracy 0.69) reported from*Lu et al. (2021b). ^*b*^*AUC, F1, and MCC for all other models reported from*Lu et al. (2021a). CNN-3Pool + DA denotes the MIL configuration combining a convolutional neural network for feature extraction, 3Pool for permutation-invariant bag-level aggregation, and data augmentation during training.

Model	AUC	Sensitivity	Specificity	F1-Score	MCC
Neuroradiologists	0.68	0.54	0.88	**0.56**	**0.58**
MI-SVM + DA	0.60 ± 0.03	-	-	0.62 ± 0.03	0.26 ± 0.05
NSK + DA	0.69 ± 0.05	-	-	0.66 ± 0.06	0.35 ± 0.10
MLP-Attention + DA	0.72 ± 0.03	-	-	0.65 ± 0.02	0.33 ± 0.06
Inception-3Pool + DA	0.77 ± 0.04	-	-	0.70 ± 0.05	0.43 ± 0.10
**CNN-3Pool + DA**	**0.82**±**0.06**	**0.76**	**0.80**	**0.78**±**0.08**	**0.46**±**0.19**

DA: data augmentation; AUC: area under the receiver operating characteristic curve; MCC: Matthews correlation coefficient; MI-SVM: multiple instance support vector machine; NSK: normalized set kernel; MLP: multilayer perceptron; CNN: convolutional neural network; F1-score: harmonic mean of precision and recall; SD: standard deviation.

Two approaches for combining information from multiple spectra were tested: a pooling strategy (3Pool) and an attention mechanism. The pooling method performed better in complex networks, whereas the attention mechanism provided advantages in simpler models. Data augmentation consistently improved performance by 3–5%.

To provide a comprehensive performance assessment beyond a single metric, we also report the F1-score, which balances sensitivity and precision, and the Matthews correlation coefficient (MCC), which reflects the overall quality of binary classification by integrating all true and false predictions. These complementary measures confirm the model's robust and balanced diagnostic performance under realistic clinical conditions. For contextual comparison, performance metrics of additional MIL architectures evaluated on the same dataset are reported in a previously published methodological study (Lu et al., 2021a).

### 3.2 Comparison with neuroradiologists

For comparison with human interpretation of MRS alone, eight neuroradiologists with varying levels of experience in ^1^H-MRS, ranging from beginners to neuroradiology specialists, evaluated a total of 844 spectra from 42 patients (approximately 105 spectra per reader). The spectra were distributed among the readers, with each reader evaluating a different subset, and were classified as tumor or non-tumor based solely on the spectral information. No structural MRI or clinical information was provided. The readers achieved a high specificity of 0.88 but a lower sensitivity of 0.54, resulting in an average F1-score of 0.56. Individual reader performance varied substantially, with sensitivities ranging from 0.325 to 0.872 and specificities ranging from 0.537 to 0.971 (Supplementary Table S1). In contrast, our model achieved a more balanced performance, with an F1-score of 0.78 (see Fig. 2).

**Fig. 2:**
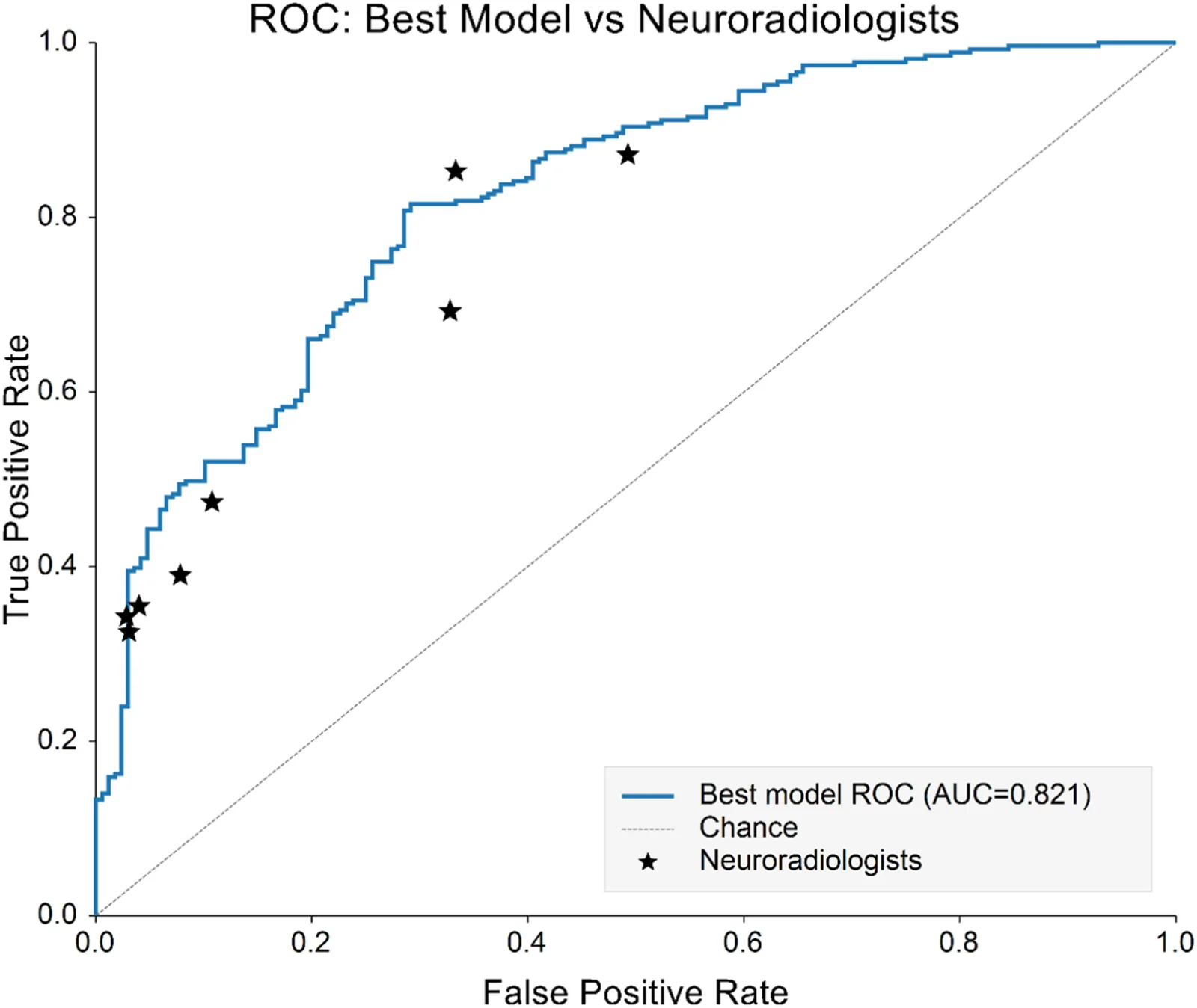
Receiver operating characteristic (ROC) curve of the best-performing MIL configuration (CNN with 3Pool aggregation and data augmentation; AUC = 0.82) and operating points of the neuroradiologists (stars). The participating readers showed an average specificity of 0.88 and sensitivity of 0.54. TPR: true positive rate (sensitivity); FPR: false positive rate (1 − specificity). Adapted from Lu et al. (Lu et al., 2021a).

### 3.3 Interpretability of the model

To investigate the interpretability of the attention-based MIL approach, attention weights generated by the Hatami-Attention model were visualized (Fig. 3) . The Hatami-Attention model achieved a patient-wise AUC of 0.80 ± 0.04 without data augmentation and 0.81 ± 0.05 with data augmentation. The attention weights indicate the relative contribution of individual spectra within a patient-level bag. In tumor spectra, higher attention weights were associated with reduced NAA (2.0 ppm) and elevated Cho (3.2 ppm), Glx (2.2–2.4 ppm), Lac (1.4 ppm), and lipid signals (0.9 ppm), whereas in non-tumor spectra, preserved NAA and a normal creatine-to-choline ratio were associated with higher attention weights. These patterns are consistent with established spectroscopic features of neoplastic and non-neoplastic brain tissue (Hattingen et al., 2009; Faghihi et al., 2017; Fan et al., 2004; Fan, 2006; Rae, 2009). Importantly, these attention weights are specific to the Hatami-Attention architecture and should not be interpreted as explaining the predictions of the best-performing CNN-3Pool + DA model.

**Fig. 3:**
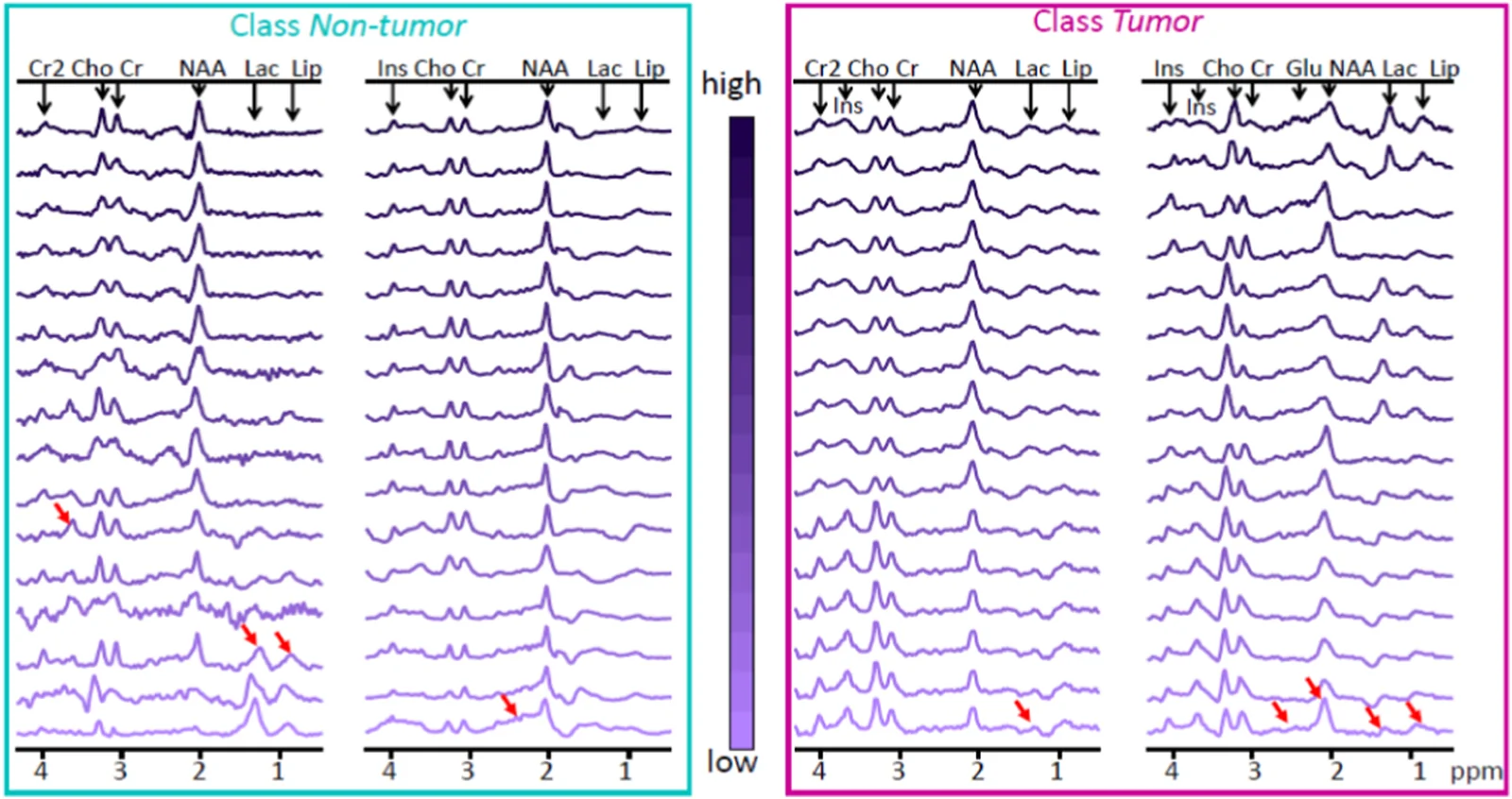
**Attention-weight visualization generated by the Hatami-Attention MIL model.** Exemplar patient-level bags of MRS spectra are shown with color-coded attention weights indicating the relative contribution of individual spectra to the bag-level representation. In tumor spectra, higher attention weights are associated with reduced NAA and elevated Cho, Glx, Lac, and lipid signals, whereas in non-tumor spectra, higher attention weights are associated with preserved NAA and normal Cr/Cho ratios. Red arrows indicate features characteristic of the opposite class. These attention weights are specific to the Hatami-Attention model and do not represent an interpretation of the CNN-3Pool + DA model. NAA: N-acetylaspartate; Cho: choline; Glx: glutamate and glutamine; Lac: lactate; Cr: creatine; Cr/Cho: creatine-to-choline ratio; ppm: parts per million. *Reproduced from Lu* et al. (Lu et al., 2021a)*, with permission.*

## 4 Discussion

### 4.1 Principal findings

This retrospective study demonstrates that a deep learning-based multiple instance learning approach can differentiate tumor from non-tumor brain lesions using proton MR spectroscopy data. Our best-performing model achieved an AUC of 0.82, an F1-score of 0.78, and an MCC of 0.46 on heterogeneous, weakly labeled clinical data. In the MRS-only reader comparison, the model achieved a sensitivity of 0.76 and a specificity of 0.80, whereas the participating readers showed an average sensitivity of 0.54 and specificity of 0.88.

Regarding the aggregation strategies, neither approach was consistently superior across the evaluated network architectures. The 3Pool approach performed better when combined with the CNN-based and Inception architectures, whereas attention-based aggregation performed better with the MLP architecture. These findings suggest that the relative performance of fixed pooling and attention-based aggregation may depend on the underlying feature-extraction architecture rather than reflecting a general advantage of either aggregation strategy.

### 4.2 Metabolic basis of model performance

Analysis of attention weights revealed that the Hatami-Attention model assigned higher attention weights to metabolic patterns consistent with established tumor biology. In neoplastic tissue, the algorithm assigned high importance to reduced N-acetylaspartate (NAA), a marker of neuronal integrity and viability, reflecting neuronal loss or dysfunction in tumor-infiltrated regions (Faghihi et al., 2017; Rae, 2009). Elevated choline (Cho) received substantial attention, corresponding to increased membrane turnover and cell proliferation characteristic of neoplastic growth (Fan et al., 2004). Similarly, increased lactate (Lac) and lipids (Lip) were prioritized, reflecting the metabolic shift toward anaerobic glycolysis (Warburg effect) and cellular necrosis commonly observed in malignant gliomas (Fan, 2006; Rae, 2009).

In non-tumor spectra, preserved or elevated NAA and normal creatine-to-choline ratios were associated with higher attention weights. Interestingly, the glutamine/glutamate (Glx) region at 2.2–2.4 ppm, which is not routinely quantified in clinical practice, received notable attention weights in both classes. These observations suggest that the attention-based model captures physiologically meaningful metabolic patterns beyond conventional metabolite ratios, potentially providing additional diagnostically relevant information.

### 4.3 Clinical implications

The comparison between the proposed model and neuroradiologists requires careful interpretation. In this study, human readers were intentionally restricted to MRS data alone to match the model input, whereas clinical decision-making typically integrates multimodal imaging and clinical context. Therefore, the observed performance differences should not be interpreted as a direct replacement of expert assessment, but rather as evidence that machine learning can effectively leverage metabolic patterns in MRS data.

The main clinical challenge addressed here is the differentiation of neoplastic from non-neoplastic T2-hyperintense brain lesions, which often present overlapping features on MRI((Hourani et al., 2008), (Krouwer et al., 1998)). MRS has demonstrated utility in this context, though with limitations (Setzer et al., 2007). Unlike conventional MRI, which depicts structural information, MRS captures the biochemical composition of tissue. However, conventional interpretation often relies on single metabolite peaks or their ratios, whereas metabolites form complex, overlapping spectral patterns that may contain additional diagnostic information. Considering the entire spectral profile as a biochemical fingerprint enables machine learning models to exploit these subtle patterns for more objective lesion classification.

Our findings suggest that combining MRS with machine learning may serve as an objective adjunct in ambiguous cases, supporting radiologists by providing reproducible metabolic assessments. The model's higher sensitivity in the MRS-only reader comparison suggests potential value in detecting tumor-associated spectral patterns that may be overlooked during isolated spectroscopic interpretation. Integration of such algorithms into clinical workflows could support less experienced radiologists or serve as a second reader in complex cases. However, the model should be viewed as a diagnostic aid rather than a replacement for expert interpretation, particularly given that clinical context, patient history, and multimodal imaging findings remain essential for comprehensive diagnosis.

### 4.4 Comparison with literature

Previous work on MRS-based tumor classification using machine learning achieved accuracies around 84% using support vector machines with manually extracted metabolite ratios (Ranjith et al., 2015). Recent approaches explored generative models for data augmentation (Olliverre et al., 2018) and semi-supervised learning (Cruz-Barbosa and Vellido, 2011). Our approach differs by processing raw spectral data without manual feature extraction, potentially capturing subtle patterns not reflected in conventional metabolite ratios.

In contrast, recent deep learning studies on conventional MRI for brain tumor classification (Bae et al., 2020; Laukamp et al., 2019) achieved comparable accuracies but required structural imaging rather than metabolic information. The integration of MRS with MRI radiomics remains underexplored.

The multiple instance learning framework addresses a fundamental challenge in medical imaging: the discrepancy between patient-level diagnoses and voxel-level measurements. This is particularly relevant in gliomas, where tumor margins are infiltrative and individual voxels may contain mixtures of tumor, peritumoral edema, and normal tissue. By aggregating information from multiple spectra per patient, our approach better reflects the clinical reality of heterogeneous lesions.

### 4.5 Methodological considerations

The multiple instance learning approach (Dietterich et al., 1997; Carbonneau et al., 2018; Campanella et al., 2019) proved essential for handling the inherent ambiguity in patient-level labeling of individual spectra. Traditional single-spectrum classification would assign all spectra from a tumor patient as ‘tumor,’ potentially mislabeling spectra from peritumoral or healthy tissue. Our bag-based approach allowed the model to learn patient-level patterns while accounting for within-patient heterogeneity.

Various approaches have been proposed (Andrews et al., 2002; Gärtner et al., 2002; Ray and Craven, 2005), with pooling methods performing best in more complex networks (CNN, Inception) (Hatami et al., 2018; Szegedy et al., 2015), whereas attention mechanisms (Ilse et al., 2018) provide interpretability through visualization of diagnostically relevant spectra and flexibility in simpler architectures.

Data augmentation (Shorten and Khoshgoftaar, 2019) improved model robustness by increasing spectral variability, and using approximately six spectra per patient offered an effective balance between accuracy and computational efficiency.

### 4.6 Limitations and future directions

Several limitations warrant consideration. First, all data, including the independent hold-out test cohort, originated from a single institution and acquisition environment using a standardized MRS protocol (3T, TE = 30 ms, PRESS sequence). Thus, the present evaluation represents independent internal hold-out validation rather than external validation. The independent hold-out cohort was completely withheld from training and validation. Nevertheless, generalizability to data acquired at other institutions and with different scanners, field strengths, echo times, acquisition protocols, or processing pipelines remains to be established. Future multicenter studies incorporating heterogeneous acquisition settings are needed to assess the generalizability and clinical applicability of the proposed approach (Kickingereder et al., 2019).

Second, our classification was binary (tumor vs. non-tumor) and did not differentiate tumor subtypes or grades according to WHO 2021 classification (Weller et al., 2022; Louis et al., 2021). While this addresses the primary clinical question of neoplastic versus non-neoplastic lesions, further refinement to distinguish glioma subtypes (astrocytoma vs. oligodendroglioma) or grades would enhance clinical utility, particularly given the prognostic and therapeutic implications of molecular markers (IDH mutation, 1p/19q codeletion) in modern neuro-oncology.

Third, the model was trained and validated exclusively on MRS data, without incorporating other imaging techniques. Integrating structural MRI, diffusion and perfusion imaging, or amino acid PET(40,41) could provide complementary anatomical and functional information, potentially improving diagnostic accuracy and helping to better characterize tumor heterogeneity. Such multimodal approaches are promising for enhancing clinical decision-making in brain tumor diagnosis.

Another limitation concerns model interpretability. Although the attention-based MIL approach allowed visualization of the relative contribution of individual spectra, these attention weights do not directly explain the predictions of the best-performing CNN-3Pool + DA model. Direct interpretability analyses of CNN-3Pool + DA, such as feature occlusion, integrated gradients, saliency analysis, or SHAP, were not performed in the present study. Future studies should apply such methods directly to the best-performing model to better characterize the spectral regions driving its predictions.

Finally, histopathological confirmation was not available for all cases. While neoplastic lesions were histopathologically confirmed, a lesion was classified as non-neoplastic if the clinical and paraclinical findings, as well as the subsequent clinical and radiological course, where available, did not support or were inconsistent with the initial suspicion of a neoplasm. In such cases, invasive neurosurgical tissue sampling was not considered justified given the associated risk–benefit profile. The requirement for histopathological verification as an inclusion criterion would have resulted in the exclusion of a considerable proportion of non-neoplastic lesions. Due to the retrospective nature of the study, detailed patient-level information on the duration and specific criteria of imaging follow-up was not systematically available. The use of different reference standards for the two groups may therefore introduce some uncertainty in the ground-truth classification.

Although 95% confidence intervals were calculated for individual reader performance, no formal statistical comparison between model and human performance was performed. Future studies using an appropriately designed reader study should include formal statistical comparisons to further assess differences in diagnostic performance.

Several research directions merit exploration. Multicenter prospective validation should assess model performance across diverse patient populations and technical parameters. Extension to tumor subtyping according to WHO 2021 classification would align the approach with current diagnostic standards and potentially guide treatment selection based on molecular profiles (Capper et al., 2018; Chang et al., 2018).

Integration of multiparametric imaging represents a particularly promising direction (Zhang et al., 1987; Pereira et al., 2018). While MRS provides metabolic characterization, structural MRI sequences (T1, T2, FLAIR) define lesion morphology and anatomical relationships, diffusion-weighted imaging reflects tissue cellularity and microstructure, perfusion imaging captures hemodynamic properties, and molecular imaging techniques such as O-(2-[^18^F]fluoroethyl)-L-tyrosine (FET) PET assess active tumor metabolism through amino acid uptake (Overcast et al., 2021). The complementary nature of these modalities suggests that multimodal deep learning models could integrate metabolic, structural, and functional information for more comprehensive lesion characterization. FET-PET is particularly valuable for distinguishing true tumor progression from treatment-related changes (pseudoprogression) and for delineating metabolically active tumor margins for surgical planning or radiation therapy targeting (Van De Weijer et al., 2022; Lohmann et al., 2019; Albert et al., 2016). Future extensions of the MIL framework could therefore incorporate regional or multimodal features, including radiomic and susceptibility-based measures, as instances or feature representations within patient-level bags, potentially providing complementary information on metabolic, structural, and tissue-composition changes.

Advanced explainability methods beyond attention visualization, such as SHAP (Shapley Additive exPlanations) analysis (Lundberg and Lee, 2017), could quantify individual metabolite contributions to classification decisions. Such approaches may enhance the interpretability and clinical acceptance of AI systems in medical imaging (Holzinger et al., 2019), potentially revealing novel diagnostic biomarkers or metabolic patterns not recognized in conventional analysis.

Beyond brain tumor classification, the patient-level MIL framework may also have potential applications in Alzheimer's disease, other neurodegenerative disorders, and normal aging. For example, spectra acquired from different anatomical regions could be represented as individual instances within a patient-level bag, allowing spatially distributed metabolic information to be integrated into a single classification framework. Such applications, as well as multimodal extensions of the framework, will require evaluation in dedicated studies (Uchida et al., 2025, 2026).

Finally, prospective clinical trials evaluating the impact of ML-assisted MRS interpretation on diagnostic accuracy, inter-reader agreement, and patient outcomes would provide evidence regarding its potential clinical utility. Such studies should assess whether algorithm assistance improves diagnostic confidence, reduces unnecessary biopsies, or accelerates treatment initiation.

## 5 Conclusion

This study demonstrates that deep learning applied to MR spectroscopy accurately differentiates brain tumors from non-neoplastic lesions (AUC 0.82), achieving a balanced trade-off between sensitivity and specificity compared to neuroradiologists. Attention-based analysis highlighted physiologically meaningful metabolic patterns, including reduced NAA and elevated choline, lactate, and lipid signals, while also identifying potentially relevant additional spectral features. Multiple instance learning effectively addresses patient-level diagnosis from heterogeneous voxel data. Multicenter validation, multiparametric imaging integration, and interpretable AI frameworks represent important next steps toward clinical implementation.

## CRediT authorship contribution statement

**Iskra Gacheva:** Writing – original draft, Visualization, Investigation, Formal analysis, Data curation. **Diyuan Lu:** Writing – review & editing, Validation, Software, Methodology, Formal analysis. **Gerhard Kurz:** Writing – review & editing, Software, Methodology, Formal analysis. **Jochen Triesch:** Writing – review & editing, Supervision, Methodology. **Elke Hattingen:** Writing – review & editing, Supervision, Conceptualization. **Nenad Polomac:** Writing – review & editing, Validation, Supervision, Conceptualization.

## Ethics declaration

The authors have NOT obtained informed consent from participants or their legal representatives. This retrospective study used previously acquired clinical MR spectroscopy data. The requirement for individual informed consent was waived by the responsible ethics committee due to the retrospective nature of the study. Patient privacy and confidentiality were protected throughout the study.

This study was performed in compliance with relevant laws, regulatory frameworks and guidelines where the research took place. This study was approved by the Ethics Committee of University Hospital Frankfurt and the Institutional Review Board of the University Cancer Center Frankfurt (UCT). (Approval No. SNO-9-2019)

## Code availability statement

The code used for model implementation and analysis is available from the corresponding author upon reasonable request.

## Declaration of generative AI use

During the preparation of this work, the authors used ChatGPT to improve language and readability. After using this tool, the authors reviewed and edited the content as needed and take full responsibility for the content of the published article.

## Funding statement

This research did not receive any specific grant from funding agencies in the public, commercial, or not-for-profit sectors.

## Declaration of competing interest

The authors declare that they have no known competing financial interests or personal relationships that could have appeared to influence the work reported in this paper.

## Data Availability

The data that has been used is confidential.
