# Supplementary material for: Multiple instance learning for differentiating neoplastic and non-neoplastic brain lesions using MR spectroscopy
Source: Neuroimage Rep. 2026 Oct 17;6(4):100418. doi: 10.1016/j.ynirp.2026.100418 (PMC13695253; doi:10.1016/j.ynirp.2026.100418)
Supplement: Supplementary file 1 [file mmc1.docx]

# Supplementary Table S1

Individual reader performance for classification of ^1^H-MR spectra as tumor or non-tumor.

| **Reader** | **Sensitivity (95% CI)** | **Specificity (95% CI)** |
| --- | --- | --- |
| 1 | 0.355 (0.211–0.531) | 0.960 (0.889–0.986) |
| 2 | 0.325 (0.201–0.480) | 0.969 (0.895–0.992) |
| 3 | 0.474 (0.325–0.627) | 0.892 (0.794–0.947) |
| 4 | 0.692 (0.536–0.814) | 0.672 (0.550–0.774) |
| 5 | 0.371 (0.232–0.537) | 0.971 (0.902–0.992) |
| 6 | 0.824 (0.665–0.917) | 0.681 (0.566–0.777) |
| 7 | 0.390 (0.257–0.543) | 0.906 (0.810–0.956) |
| 8 | 0.872 (0.733–0.944) | 0.537 (0.419–0.651) |

CI, confidence interval. 95% confidence intervals were calculated using the Wilson method.

**Supplementary Table S2**

Composition of the five patient-level cross-validation test folds in the development cohort.

| **Fold** | **Patients** | **Tumor patients** | **Non-tumor patients** | **Spectra** | **Tumor spectra** | **Non-tumor spectra** |
| --- | --- | --- | --- | --- | --- | --- |
| 0 | 75 | 54 | 21 | 1,307 | 831 | 476 |
| 1 | 75 | 52 | 23 | 1,200 | 780 | 420 |
| 2 | 75 | 44 | 31 | 1,192 | 603 | 589 |
| 3 | 75 | 43 | 32 | 1,343 | 681 | 662 |
| 4 | 83 | 51 | 32 | 1,556 | 862 | 694 |
| Total | 383 | 244 | 139 | 6,598 | 3,757 | 2,841 |

Patients were randomly permuted before partitioning; fold assignment was not stratified.
